# Feasibility of Barley Straw Fibers as Reinforcement in Fully Biobased Polyethylene Composites: Macro and Micro Mechanics of the Flexural Strength

**DOI:** 10.3390/molecules25092242

**Published:** 2020-05-10

**Authors:** Ferran Serra-Parareda, Fernando Julián, Eduardo Espinosa, Alejandro Rodríguez, Francesc X. Espinach, Fabiola Vilaseca

**Affiliations:** 1 LEPAMAP+PRODIS research group, University of Girona, Maria Aurèlia Capmany, 61, 17003 Girona, Spain; fernando.julian@udg.edu (F.J.); francisco.espinach@udg.edu (F.X.E.); 2 Chemical Engineering Department, Bioagres Group, Universidad de Córdoba, 14014 Córdoba, Spain; a.rodriguez@uco.es; 3 Advanced Biomaterials and Nanotechnology, Dept of Chemical Engineering, University of Girona, 17003 Girona, Spain; fabiola.vilaseca@udg.edu; 4 Department of Industrial and Materials Science, Chalmers University of Technology, SE 412 96 Gothenburg, Sweden

**Keywords:** barley straw, composite, flexural strength, biobased polyethylene

## Abstract

Awareness on deforestation, forest degradation, and its impact on biodiversity and global warming, is giving rise to the use of alternative fiber sources in replacement of wood feedstock for some applications such as composite materials and energy production. In this category, barley straw is an important agricultural crop, due to its abundance and availability. In the current investigation, the residue was submitted to thermomechanical process for fiber extraction and individualization. The high content of holocellulose combined with their relatively high aspect ratio inspires the potential use of these fibers as reinforcement in plastic composites. Therefore, fully biobased composites were fabricated using barley fibers and a biobased polyethylene (BioPE) as polymer matrix. BioPE is completely biobased and 100% recyclable. As for material performance, the flexural properties of the materials were studied. A good dispersion of the reinforcement inside the plastic was achieved contributing to the elevate increments in the flexural strength. At a 45 wt.% of reinforcement, an increment in the flexural strength of about 147% was attained. The mean contribution of the fibers to the flexural strength was assessed by means of a fiber flexural strength factor, reaching a value of 91.4. The micromechanical analysis allowed the prediction of the intrinsic flexural strength of the fibers, arriving up to around 700 MPa, and coupling factors between 0.18 and 0.19, which are in line with other natural fiber composites. Overall, the investigation brightness on the potential use of barley straw residues as reinforcement in fully biobased polymer composites.

## 1. Introduction

The agri-food industry is becoming increasingly important in the world. In 1950, the world population was estimated to be around 2.6 billion people according to United Nations. Seventy years later, this number is still rising (7.7 billion) and is expected to reach 10 billion by 2050 [1]. This enormous increase in population brings with it major challenges to be faced, two of which are: to provide food, and to reduce as much as possible the depletion of natural resources. In addition, socially, in recent years there has been a change in the way of life, with an increase in the population in the cities, to the detriment of rural areas, leading to depopulation that undoubtedly affects the natural environment.

The agri-food activity becomes one of the pillars on which to sustain an economic model and sustainable development, environmentally, economically, and socially. If society really wants to approach a sustainable development, it is necessary to leave the linear economic model and evolve to a circular one, where each of the inputs is valued, so that the amount of waste tends to zero. In the agricultural activity the great amount of resources that are used, human and material, do it not only in the growth of the grain or fruit, but also in the growth of the plant. This therefore generates a considerable amount of waste, also called lignocellulosic biomass, the recovery of which would bring great benefits to the agricultural economic cycle, which is sometimes in need of subsidies. In fact, if a product with added value is obtained from a waste, an economic return can be obtained from it.

World cereal production in 2018 was 2,968 MM tons, with a cultivated area of 728 MM hectares. Barley contributed 4.75% of total production, with 141 million tons, representing production in the countries of the European Union a 40% (56 million tons), according to the FAOSTAT (Food and Agriculture organization of the United Nations). It can be deduced, considering a straw/grain ratio around 1 [2,3], the enormous amount of waste that this activity generates every year.

Using a byproduct from any agri-food or industrial process to obtain products with added value is one of the goals of the circular economy and it is also in line with the principles of green chemistry [4]. In some cases, cereal straws are left in the fields to be incinerated or decomposed as fertilizer for the next harvest [5]. These practices provide undoubted benefits but also produce CO_2_ emissions and can be impractical for long straws and useful only for stubble. Moreover, country regulations are increasingly controlling agri-food waste incineration in order to prevent fires and unhealthy emissions. Thus, other solutions to manage such agri-food must be explored. In the case of barley straw there have been intents to use such waste as biofuel source [6,7,8,9] with successful results. Nonetheless, the use of this waste as biofuel source is only possible if a treatment plant is near enough in terms of transport costs. There is also literature dealing with the use of barley straws in the paper and board industry [10,11]. Other researchers have proposed barley straws for algae control purposes [12,13] and to prevent soil erosion on some plantations [14,15]. Thus, barley straws have showed that it is possible to create value from such wastes.

Composite materials reinforcement is a field were the exploitation of lignocellulosic waste has been extensively explored [16,17]. The use of a variety of agri-food waste from annual plants as composite reinforcements has revealed the potential of such fibers as strength and stiffness enhancers [18,19,20]. Lignocellulosic reinforced materials are intended to be greener than glass fiber reinforced ones, while showing similar mechanical to be commercially competitive. The main obstacles in obtaining comparatively high strengths and stiffness with lignocellulosic fibers are, on the one hand the compatibility between hydrophobic polymer matrices and hydrophilic natural fibers that hinder obtaining strong interfaces [21,22]. On the other hand, the intrinsic properties of natural fibers are lower than those of mineral ones [23,24]. The literature shows how the use of coupling agents allows obtaining strong interfaces for polyolefin-based materials, specifically maleic anhydride-grafted polymers [22,25,26]. Thus, in the case of polyolefin-based composites, a careful dosage of coupling agent solves strong interfaces issues. The intrinsic properties of natural fibers are notably lower than glass fiber. Moreover, the properties of natural fibers show higher scatter than manmade materials. Thus, it is not possible to obtain the same strengths at the same reinforcement contents. Nonetheless, it is possible to add higher amounts of natural fiber to a composite than glass fiber and obtain similar mechanic properties [27,28].

Surprisingly, the literature about barley straw reinforced polymers is scarce. Barley straws are mainly used as concrete or elastomer fillers [29,30,31,32]. Hyvärinen and Kärki explored using barley straw instead of wood fibers as polypropylene reinforcement [33]. The researchers found how the mechanical properties of barley straw reinforced materials were lower than wood fiber reinforced ones. Silva-Guzman et al. researched the effect of barley straw on the mechanical properties of a corn starch polymer-based composite [34]. The authors observed a positive effect of the presence of the reinforcements on the strength and stiffness of the materials. Nonetheless, the authors used low reinforcement contents, with a 15% *w*/*w* highest percentage. Rojas-Leon et al. used barley straw particles with recycled high-density polyethylene (HDPE) to obtain particleboards [35]. In this paper the interface between barley straw and HDPE was weak as the mechanical properties of the materials decreased with the filler contents. Serra-Parareda et al. researched the effect of barley straw content on the tensile strength of mold injected composites [36]. In this paper the authors found that adding a 6% of coupling agent returned the highest tensile strength values. The authors also obtained the intrinsic tensile strength and Young’s modulus of the reinforcements. To the extent of authors’ knowledge there is no literature on the flexural strength of barley straw reinforced polyolefin composites.

Knowing the flexural properties of a material is of great importance for engineers. Moreover, when the material is clearly anisotropic, as semi-oriented short fiber reinforced composites [37,38,39]. Usually, products and components are used under bending conditions and purely tensile cases are scarce in comparison. Thus, designers are interested in previewing the behavior of such components under flexural loads [40,41]. Additionally, the intrinsic flexural strength of barley straw is unknown in the literature. Knowing such value can be used to model the behavior of composites at different reinforcement contents.

In the current investigation barley straw fibers were submitted to elevated temperatures and then defibrated to obtain single fibers. Fully biobased composites were prepared based on a biobased polyethylene matrix reinforced with 15, 30 and 45 wt.% of barley fibers. A coupling agent was added to the formulation to enhance the interfacial adhesion. The materials were injection-molded and subjected under three-point bending test to evaluate the flexural properties. The properties were studied from a macro and micromechanical viewpoint, where the intrinsic flexural strength of the fibers, the coupling factors, and the contribution of the reinforcements to the flexural strength of the composite were assessed as main important outcomes. Overall, the current investigation explores the potential of barley straw residues in added value applications by its incorporation in a fully biobased matrix, contributing to global sustainable development.

## 2. Results

### 2.1. Fibers Characterization

Barley straws were submitted to steam-water treatment with further defibration by means of Sprout Waldron equipment, obtaining barley thermomechanical (TM) fibers. The chemical composition and morphology of the fibers was examined as two main important factors affecting composite’s properties. On the one hand, the chemical composition of the fibers plays a key role in establishing the extend of interaction between the fibers and the matrix, assisted by the coupling agent. This phenomenon will affect the stress-transfer between the phases inside the composite [42,43]. On the other hand, a definite fiber aspect ratio is required for the effective stress-transfer between the phases. In this way, when the stress concentration at the fiber ends, this leads to the matrix cracking. Thereby, shorter aspect ratios will bring to more fiber ends, acting as stress concentration points with failure potential [44].

**Figure 1:**
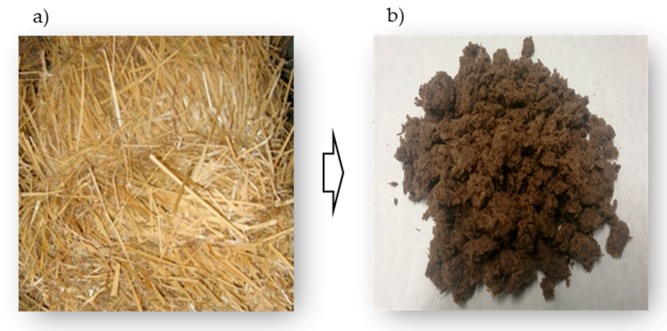
Barley straw images (**a**) before being treated and (**b**) after the thermomechanical process.

Hence, the initial evaluation of the chemical and morphological composition is needed. Table 1 presents the chemical constituents and the mean fiber length and diameter of the original barley straw and the thermomechanical fibers. For readers’ convenience, illustrations of untreated barley straw and thermomechanically treated barley fibers are presented in Figure 1.

From Table 1, barley straw is rich in holocellulose with a relatively small portion of lignin in comparison with other sources of natural fibers. For example, wood fibers possess higher lignin content, with minor amount of holocellulose. This is explained by the fact that in wood fibers lignin is needed to ensure the maintenance of the fiber cell wall structure [45,46]. The thermomechanical treatment removed part of the lignin, some of the extractives and ashes. As expected, an increase in the carbohydrate content (holocellulose) was experimented owing to changes of the lignin, extractives, and ashes content. The thermomechanical treatment also promoted the release of fiber elements with high aspect ratio (38.0). The weighted fiber length is here considered.

By treating the fibers at high temperatures, the lignin is softened, and fibers breakage is more likely to occur at the outsider layers of the fiber cell wall, between the primary wall and middle lamella. Here is where the largest concentration of lignin (~70 wt.%) is found, attaching the individual fibers together, with minor amounts of cellulose (~10 wt.%) and hemicellulose (~20 wt.%) [45].

**Figure 2:**
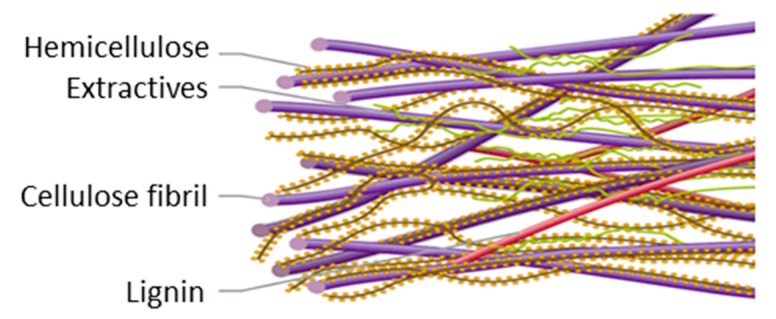
Illustration of the distribution of the lignin and carbohydrates in the fiber surface.

During the thermomechanical treatment, part of the lignin can be dissolved in the hot water and released from the fiber cell wall during the mechanical defibration. Lignin is bonded to the surface of carbohydrates (Figure 2), therefore its removal can finally lead to the release of hemicelluloses, extractives, and inorganic matter. As a result, the global yield in thermomechanical processes renders values between the 85% and 95% depending on the severity of the treatment, indicating the loss of the chemical constituents throughout the process [47,48].

Overall, the thermomechanical fibers produced from barley straw show high amount of holocellulose fibers with relatively high aspect ratio. Therefore, in regions with big availability of this biomass, deforestation can be prevented. These fibers show to be good candidates as reinforcing fibers in composite materials.

### 2.2. Optimization of the Coupling Agent

The flexural properties in composite materials depend on the type and amount of reinforcement, orientation and morphology of the fibers, the dispersion of the reinforcement inside the matrix, and largely on the quality at the interphase [5,37,49]. However, the different nature of natural fibers and thermoplastics hinders the spontaneous interactions between both materials. The lack of compatibility is explained by the different chemical structure of thermoplastics and natural fibers driving to different polarities. The hydroxyl groups in the fiber surface gives them and hydrophilic nature, whereas the hydrocarbon structure of thermoplastics confers them hydrophobicity.

**Figure 3:**
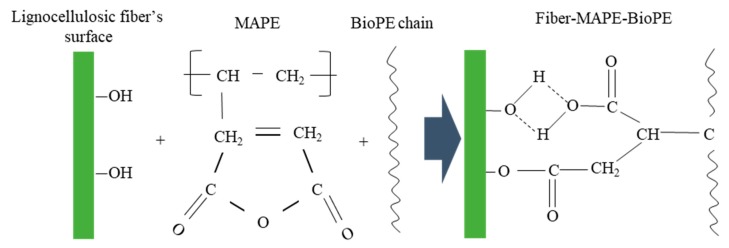
Illustration of maleic anhydride polyethylene (MAPE) interaction between the fiber and the matrix.

As a result, the poor compatibility hinders the stress-transfer capacity and makes difficult the increment of the strength by the addition of the lignocellulosic reinforcement. To enhance the interfacial adhesion, coupling agents have proved to work efficiently in this purpose. More specifically, maleic anhydride polyethylene (MAPE) can be used to increase the interactions between both phases. In this context, the coupling agent form linkages with the hydroxyl groups in the fibers’ surface by means of hydrogen bonds and covalent interaction with the maleic groups, and by chain entangling with the unmodified BioPE chains, as illustrated in Figure 3.

The efficiency of the coupling agent depends largely on the amount of bonding and the interaction quality with the natural fibers [50,51]. The optimal content of MAPE in natural fiber composites has been found to be between 4 and 8 wt.% with respect to fiber content [42,52,53]. The amount of MAPE added will depend on the fiber content, thus, the optimal amount of MAPE needed to enhance the interfacial bonding will be investigated in view of the fiber loading.

**Figure 4:**
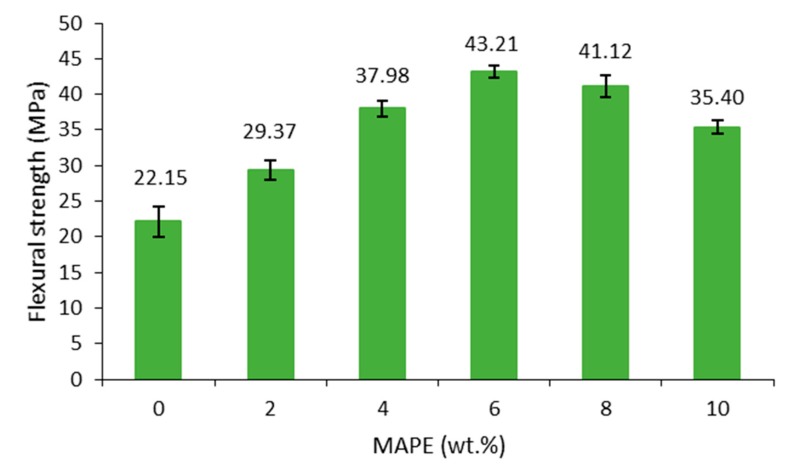
Flexural strength of composites at 30 wt.% and different MAPE content.

To investigate how the content MAPE affected the interfacial adhesion, varying amounts of MAPE (0, 2, 4, 6, 8, and 10 wt.%) with respect to fiber content were added to composites reinforced with 30 wt.% of barley fibers. The coupling agent was optimized to achieve the highest flexural strength, indicative of an optimal fiber-to-matrix interfacial union. When the amount of coupling agent was optimized, the same MAPE percentage was then applied to the rest of the composites with different fiber loadings. These results are shown in Figure 4.

The composite material without MAPE showed a similar flexural strength than the neat matrix (21.25 MPa), evidencing scarce compatibility between composite phases. Still, however, the addition of barley TMP fibers into the polymer did not decrease the flexural strength. However, by adding the coupling agent the flexural increases, reaching a maximum value at 6 wt.% of MAPE. For lower amounts of coupling agent, little improvement was observed, whereas much high amounts of coupling agent the gaining in property was again reduced. The reduction of the flexural strength at too high amounts of coupling agent can be attributed to the much shorter polymer lengths of MAPE polymer, as compared to the polymer itself; the benefits of the coupling agent were less compared to the effect of shorter polymer chains in the formulation.

Once the content of MAPE was optimized, the flexural properties of the composite materials at other formulations were examined.

### 2.3. Flexural Properties of Barley Fiber Composites

The barley fibers were incorporated to a biobased polyethylene, and the flexural properties measured. The results of the bending test as function of the fiber loading are presented in Table 2, where V^f^ is the reinforcement volume fraction, σ_f_^c^ is the flexural strength of the composite, **ε_f_^c^** is the deformation at the maximum flexural strength value, and σ_f_^m^* is the contribution of the matrix to the tensile strength.

**Figure 5:**
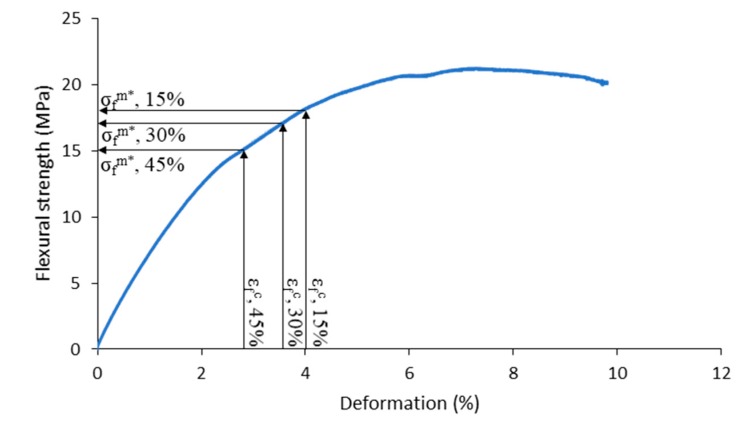
Flexural stress-strain curve of BioPE. Evaluation of the matrix contribution to the flexural strength of the composite.

The values of σ_f_^m^* were obtained from the stress-strain curves of the neat matrix by computing the stress of the matrix at the deformation where the maximum stress of the composite was produced (Figure 5).

The flexural strength of the composites followed a linear evolution with the fiber volume fraction. This indicated a proper stress transfer between the phases and a good dispersion of the reinforcement inside the plastic matrix. The addition of the fibers produced an enhancement in the flexural strength about the 42%, 103% and 147% in the composite reinforced with the 15, 30 and 45 wt.%, with respect to the neat matrix.

These are remarkable increments considering the type of raw biomass used, which is an agricultural residue. In fact, barley composites exhibited comparative flexural properties than other natural fiber composites by using wood fibers, such as spruce, and higher than other agricultural residues [54,55,56]. This performance can be attributed to the chemical composition of barley fibers.

**Figure 6:**
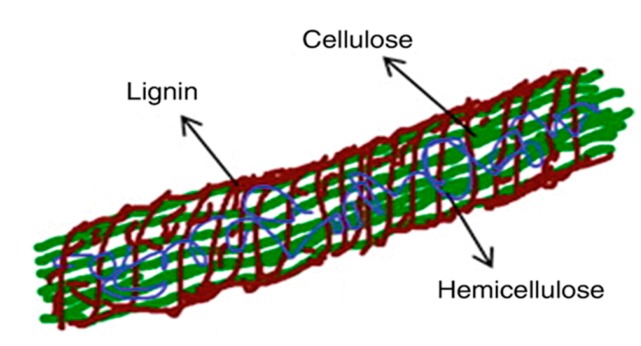
Hierarchical structure of lignocellulosic biomass.

Cellulose is the major crystalline compound and its aligned structure confers the strength and stiffness to the fiber cell wall structure. As a result, one can expect a higher contribution to the flexural strength of the composite when the reinforcement possesses higher amounts of holocellulose [57]. Besides, lignin is an amorphous polymer with a certain degree of hydrophobicity, which does not significantly contribute to the mechanical properties of the fibers, though, the compound plays a major role in binding the cellulosic chains and favoring the stress-transfer within the fibers and with the matrix [58] (Figure 6). According to Bledzki et al. [59,60], an increment on the composite’s strength can be attributed to higher cellulose and lignin content, as well as to an optimal dispersion and interfacial adhesion of the reinforcement with the matrix. Moreover, Shebani et al. [61] stated that optimal amounts of lignin can act as binding between the cellulose fibrils, granting to the stress transfer between the fibrils. This statement is in accordance with previous investigation of the research group dealing with the influence of lignin in natural fiber composites [45].

In the present case, fibers’ breakage occurred at the outer layers of the fiber cell wall during the thermomechanical treatment, where the major concentration of lignin is placed. The outer layers are covered in its surface by lignin, and it is there where one can hope for an optimal compatibility fiber-to-fiber and fiber-to-matrix, favoring the stress-transfer throughout the fibers. The fact that the BioPE can be reinforced up to a 45 wt.% of these fibers is explained by the good compatibility given by the chemical composition of the fibers.

Apart from its chemical composition, the high aspect ratios of the fibers also confer the material a larger capacity of transferring the stress through the fibers and incrementing the final strength of the material.

The deformation of the materials was significantly affected by the addition of a more rigid phase. This fact is attributed to the increased adhesion between the phases and the greater rigidity of barley fibers in comparison with the soft BioPE [41,62]. This reduced the deformation ability of the material. A micro-mechanical analysis was also performed to better understand the behavior of the composites.

### 2.4. Intrinsic Flexural Strength Properties

The strength of natural fiber composites is a combination of the strength supported by the polymeric phase and the stress effectively transferred to the reinforcing fibers. As abovementioned, the stress supported by the polymeric phase is obtained from the stress-strain curve of the neat matrix. Thereby, the difference between the strength of the composite and the stress supported by the plastic matrix is attributed to the stress transferred to the reinforcement. Thereafter, it is possible to quantify the effectiveness of the fibers inside the composite, as well as its intrinsic mechanical properties.

One of the simplest methods used to express the contribution of the phases to the material’s strength is by using the modified Rule of Mixtures (mRoM) [63,64]. The model was initially developed to be applied to tensile properties, though, it can also be extended to flexural ones. The mRoM for tensile and flexural properties are shown in Equations (1) and (2), respectively.

(1) $$\begin{array}{cc} \mathrm{Tensile}\mathrm{mRoM} & \sigma_{t}^{c}=f_{c,t}\times \sigma_{t}^{F}\times V^{f}+\sigma_{t}^{m^{*}}\times \left(1-V^{f}\right) \end{array}$$

(2) $$\begin{array}{cc} \mathrm{Flexural}\mathrm{mRoM} & \sigma_{f}^{c}=f_{c,f}\times \sigma_{f}^{F}\times V^{f}+\sigma_{f}^{m^{*}}\times \left(1-V^{f}\right) \end{array}$$

where σ_t_^F^ and σ_f_^F^ are the intrinsic tensile and flexural strength of the fibers, and f_c,t_ and f_c,f_ are the tensile and flexural coupling factors. Generally, in short semi-aligned fiber composites with strong interfacial adhesion, the coupling factor tends to a value between 0.18 and 0.20. In its current shape, the mRoM contain two incognita, which are the intrinsic strength and the coupling factor.

The value of the intrinsic tensile strength of the fibers was calculated in previous works [36] by using the Kelly and Tyson modified equation and its solution, provided by Bowyer and Bader [65,66]. In that work, a pre-evaluation of the tensile properties in view of the fiber orientation, fiber morphology and interfacial adhesion was carried out. The investigation allowed the acquisition of the orientation factor (0.309) and interfacial shear strength (10.49), as important outcomes. At a 6 wt.% of MAPE, the intrinsic tensile strength of barley fibers at a 30% of reinforcement was 521.2 MPa. Though, the current investigation incorporates the tensile properties of composites reinforced with a 15 and 45 wt.%. By following the same methodology, the intrinsic tensile strength of the fibers was obtained, with values of 532.9 and 500.5 MPa, at a 15 and 45 wt.%, respectively. Once computed the intrinsic tensile strengths, one can calculate the tensile coupling factors from Equation (1) at each fiber loading.

Nonetheless, the calculus of the intrinsic flexural strength is not as straightforward as one could expect. For example, Hashemi [67] proposed a correlation between the composite’s and fiber’s tensile and flexural strength, defined by $\sigma_{f}^{F}=\left(\sigma_{f}^{c}/\sigma_{t}^{c}\right)\times \sigma_{t}^{F}$ However, as reported by the same author, this assumption may not be necessarily correct.

Recent work methodologies suggested to only account for the fiber contribution to the composite strength. A correlation was established between the contribution of the fibers to both the tensile and flexural strength of the composite, and the intrinsic flexural and tensile strength of the reinforcement. This assumption is made upon the fact that the tensile and flexural coupling factors are in the same order of magnitude, since the factor is not dependent on the type of test conducted, either flexural or tensile. Additionally, the tensile coupling factor (fc,t) and the flexural coupling factor (fc,t), which largely depend on the quality at the interphase, fiber’s morphology and dispersion of the fibers inside the matrix, should acquire alike values in both tests. Assuming this hypothesis, the net contribution of the fibers to the tensile ($f_{c,t}\times \sigma_{t}^{F}\times V^{f}$) and flexural ($f_{c,f}\times \sigma_{f}^{F}\times V^{f})$ strength of the composite should be directly correlated to the intrinsic tensile strength ($\sigma_{t}^{F}$) and intrinsic flexural strength ($\sigma_{f}^{F}$) of the fibers [38,39,68].

The global contribution of the fibers to the tensile and flexural strength of the composite can be obtained by reorganizing the mRoM. Thereby, it is possible to isolate the net contribution of the fibers to the strength of the composite with the fiber volume fraction. Afterwards, if the net contribution is plotted versus the volume fraction in each of the composites, the fiber flexural strength factor (FFSF) (Equation (3)) and the fiber tensile strength factor (FTSF) (Equation (4)) is obtained from the slope of the line [69].

(3) $$\begin{array}{cc} \mathrm{FFSF} & f_{c,\;f}\times \sigma_{f}^{F}=\left(\frac{\sigma_{f}^{c}-\sigma_{f}^{m^{*}}\times \left(1-V^{f}\right)}{V^{f}}\right) \end{array}$$

(4) $$\begin{array}{cc} \mathrm{FTSF} & f_{c,\;t}\times \sigma_{t}^{F}=\left(\frac{\sigma_{t}^{c}-\sigma_{t}^{m^{*}}\times \left(1-V^{f}\right)}{V^{f}}\right) \end{array}$$

Knowing the intrinsic tensile strength, and the global contribution of the fibers to the tensile and flexural strength of the composite, it is possible to calculate the intrinsic flexural strength of the fibers following Equation (5).

(5) $$\frac{\sigma_{f}^{F}}{\sigma_{t}^{F}}=\frac{\mathrm{FFSF}}{\mathrm{FTSF}}$$

To compute the contribution of the fibers to the tensile strength of the composite, the tensile properties are needed (Table 3). The properties were extracted from the previous work dealing with tensile properties [36].

**Figure 7:**
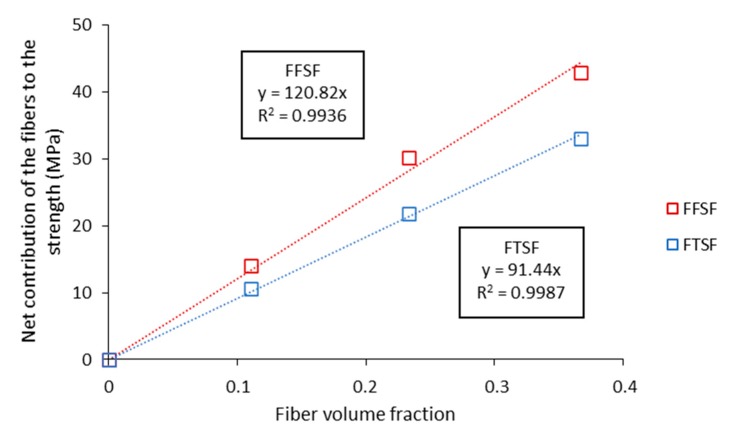
Fiber tensile strength factor (FTSF) and fiber flexural strength factor (FFSF).

Briefly, the tensile strength followed a linear evolution with the fiber content. Increments in the tensile strength parameter were obtained about the 40%, 92% and 139%. The global contribution of the fibers to the composite strength computed by means of the FTSF and FFSF are presented in Figure 7.

**Figure 8:**
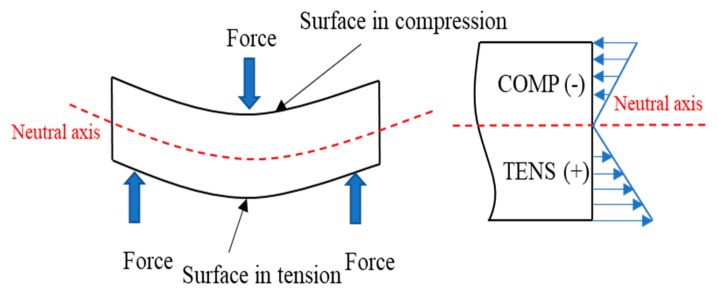
Combination of compression and tension forces during the flexural test.

The contribution of the fibers to the flexural strength (FFSF = 120.8) was significantly higher than in the tensile one (FTSF = 91.44). This is attributed to the fact that composites subjected to flexural loads support a combination of compressive and tensile forces at the cross-sectional area of the specimens (Figure 8).

Some authors explain that while composites subjected to tensile test are fully loaded under tensile stresses, flexural specimens are loaded under compressive and tensile forces at the same time. Since most of the thermoplastics have a larger capacity to withstand the load under compression rather than tensile, the part of the specimen subjected to compression is expected to contribute more than the one submitted to tensile stress. As a result, flexural specimens will support higher stresses than tensile ones. Other authors state that the anisotropy of the fibers and their semi-alignment inside the plastic can contribute more extensively to the flexural strength [56].

Overall, the FFSF was found to be higher than in other composites reinforced with different sources of agricultural residues, reflecting the potential of barley straws in composites field. In comparison with wood fiber reinforced composites, the FFSF did not differ much, though, larger discrepancies could be observed with the FTSF. Nonetheless, this could be an advantage for composite materials subjected to flexural loads since the replacement of agricultural residues for wood fibers would be an attractive alternative.

Considering the relationship between the contribution of the fibers to the flexural and tensile strength of the composite (FFSF/FTSF), and with knowledge of the intrinsic tensile strength of the fibers, it is therefore possible to determine the intrinsic flexural strength according to Equation (5). Then, by using the mRoM for both the tensile and flexural properties, the respective coupling factors can be obtained and compared (Table 4).

The intrinsic flexural strength increased to 703.4 MPa at a 15 wt.% of reinforcement, being lower at the 45 wt.% (660.7 MPa). The followed methodology was proved to work efficiently owing to the great similarities between the tensile and flexural coupling factors. As previously mentioned, the coupling factor in natural fiber composites with optimal interfaces is between 0.18 and 0.20, proving the good interface in barley composites.

## 3. Materials and Methods

### 3.1. Materials

Composite materials were prepared using biobased polyethylene (BioPE) as polymer matrix and barley straw residues as reinforcement. BioPE was kindly supplied by Braskem (Sao Paulo, Brazil). BioPE is obtained from bioethanol coming from sugarcane feedstocks. Thereby, the polymer is completely biobased and 100% recyclable in the same chain established for the conventional fossil-based polyethylene. The melt flow index of the polymer is 20 g/10 for hammer weight of 2.16 kg, with a density of 0.955 g/cm^3^. Maleic anhydride polyethylene was added as coupling agent to enhance the interfacial adhesion between the matrix and the reinforcement. The coupling agent (Fusabond MB100D) was supplied by DuPont (Wilmington, DE, USA). Barley straws residues were kindly provided by Mas Clarà S.A. (Girona, Spain). The length of a single barley straw ranged from 5 to 50 cm, with diameters between 0.1 and 0.6 cm.

Ethanol (95 wt.%), toluene (99.5 wt.%) and sulfuric acid (72 wt.%) were employed for the chemical characterization of the fibers. All reagents used in the present investigation were supplied by Sigma-Aldrich and used as received.

### 3.2. Methods

#### 3.2.1. Thermomechanical (TM) Barley Straw Fiber Production and Characterization

Barley straw was chopped by means of a blade mill with a 3 mm mesh. Straw particles were then subjected to a thermomechanical treatment for the extraction of single fibers (TMP fibers). For this, the lignocellulosic material was submitted to steam-water treatment in a pressurized reactor at 160 °C temperature and solid to liquid ratio of 1:6 for 15 min. Afterwards, the obtained suspension was filtered and washed thoroughly with distilled water. The obtained pulp was mechanically defibrated by using Sprout Waldon equipment, responsible of the fiber defibering. Finally, fibers were oven-dried at 80 °C until constant weight.

The chemical composition and morphology of the fibers was examined. The size distribution analysis was carried out using MORFI equipment (TechPAP, Gières, France). A minimum of 4 samples were analyzed, taking 30,000 images of fibers in each analysis. The analysis of the chemical constituents was carried out from the analysis of the ethanol soluble extractives (TAPPI T204 cm-07), ashes (ISO 2144:2019) and lignin (ISO/DIS 21436). The holocellulose content (cellulose + hemicelluloses) was measured by difference.

#### 3.2.2. Composites Preparation and Sample Obtaining

BioPE and barley TMP fibers were blended at weight ratios of 85/15, 70/30 and 55/45 (matrix/reinforcement) by means of an intensive Gelimat kinetic mixer. Initially, the fibers were introduced in the mixer at a speed of 300 rpm. The polymer and the coupling agent were then added to the mixer chamber maintaining constant speed. The speed was then increased up to 2500 rpm until the polymer was completely melted. The composite is then after discharged and cooled down and pelletized using a blade mill equipped with a 5 mm mesh. The material was oven-dried until constant weight.

The specimens for the flexural test were produced with a steel mold in an injection molding machine Aurburg 220 M 350-90U (Aurburg, Loßburg, Germany). Tensile specimens were also acquired for the determination of the tensile properties of the composites.

#### 3.2.3. Mechanical Test

Prior to testing, specimens were placed in a conditioning chamber (Dycometal, Sant Boi de Llobregat, Spain) at 23 °C and 50% relative humidity for 48 h, according to ASTM D618 standard. Flexural properties of the specimens were determined by means of an INSTRON universal testing machine equipped with a 5 kN load cell. The flexural test was performed following ASTM D790. Tensile properties were also measured following ASTM D638 standard. At least five specimens of each composite formulation were tested.

**Figure 9:**
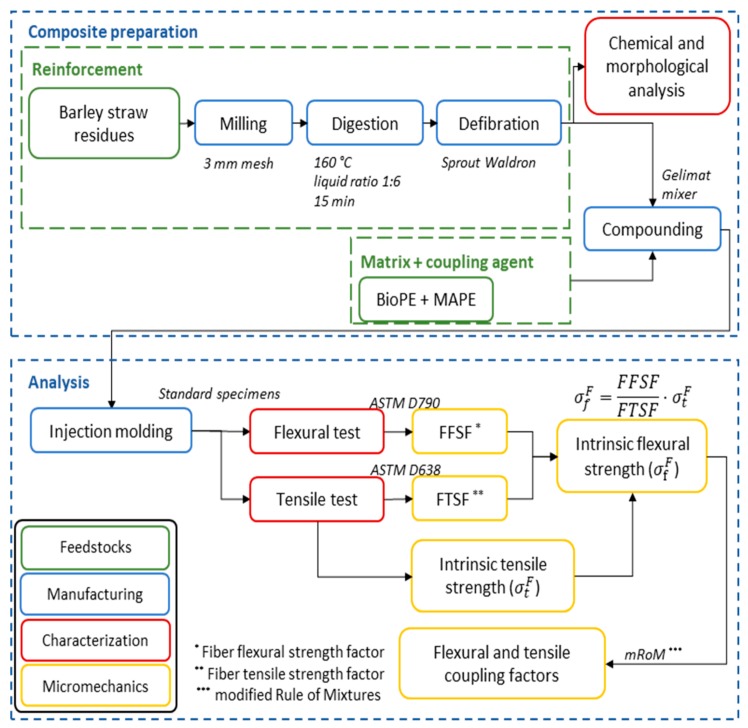
Flowchart of the current investigation.

Figure 9 presents a schematic flowchart of the experimental procedure, including composite’s preparation and the analysis of its properties.

## 4. Conclusions

The present work evaluates the feasibility of incorporating barley straw fibers as reinforcement in a biobased polyethylene to develop a fully biobased and 100% recyclable material. Barley straw was treated by means of a thermomechanical process and the resulting fibers were evaluated in terms of its chemical composition and its morphology. The efficiency of barley fibers was enhanced by the addition of anhydride maleic polyethylene as coupling agent. The flexural behavior of the material was investigated as important property determining the suitability of the material for several applications. The addition of barley straw fibers caused enlargement in the flexural strength about the 42%, 103% and 147% at 15, 30 and 45 wt.% fiber content, respectively.

A methodology was followed to determine the intrinsic flexural strength of the fibers. The methodology assumes that the flexural and tensile coupling factors are in the same order of magnitude. The coupling factors were found to be in the range from 0.18 to 0.20, an indication of the existence of strong interfaces for semi-aligned short fiber reinforced composites. The intrinsic flexural strength of barley straw changed with the amount of reinforcement, showing values ranging from 700 MPa at a 15 wt.% to 660 MPa at a 45 wt.% reinforcement content. The results from the study show the suitability of barley straw biobased composites for semi-structural and engineering purposes.

## Figures and Tables

**Table 1:** Chemical and morphological composition of barley straw and barley thermomechanical (TMP) fibers.

Composition/Morphology	Barley Straw	Barley TMP Fibers
Holocellulose (wt.%)	70.12 ± 0.54	77.67 ± 0.61
Klason lignin (wt.%)	16.45 ± 0.34	15.30 ± 0.46
Extractives (wt.%)	5.90 ± 0.76	2.73 ± 0.12
Ashes (wt.%)	7.1 ± 0.2	4.3 ± 0.3
Length ^1^ (μm)	-	745 ± 21
Diameter (μm)	-	19.6 ± 0.6
Aspect ratio (length/diameter)	-	38.0

^1^ Length weighted in length.

**Table 2:** Flexural properties of BioPE composites reinforced with barley fibers.

Sample	Reinforcement (wt.%)	V^f^	σ_f_^c^ (MPa)	ε_f_^c^ (%)	σ_f_^m^* (MPa)
BioPE	0	0	21.25 ± 0.95	7.18 ± 0.41	21.25
BioPE/Barley fibers	15	0.111	30.21 ± 1.23	4.03 ± 0.28	18.21
	30	0.233	43.21 ± 0.89	3.52 ± 0.31	16.98
	45	0.367	52.45 ± 1.45	2.85 ± 0.19	15.14

**Table 3:** Tensile properties of BioPE composites reinforced with barley fibers.

Sample	Reinforcement (wt.%)	V^f^	σ_t_^c^ (MPa)	ε_t_^c^ (%)	σ_t_^m^* (MPa)
BioPE	0	0	18.05 ± 0.74	12.18 ± 0.34	18.05
BioPE/Barley fibers	15	0.111	25.21 ± 0.64	7.65 ± 0.24	16.37
	30	0.233	34.70 ± 0.90	6.45 ± 0.31	16.76
	45	0.367	43.10 ± 0.57	4.69 ± 0.33	15.86

**Table 4:** Intrinsic flexural (σ_f_^F^) and tensile strength (σ_t_^F^) of the fibers, and flexural (fc,f) and tensile (fc,t) coupling factors.

Sample	Reinforcement (wt.%)	$\frac{FFSF}{FTSF}$	Tensile		Flexural	
			σ_t_^F^ (MPa)	fc,t	σ_f_^F^ (MPa)	fc,f
BioPE + barley	15	1.32	532.9	0.18	703.4	0.18
	30		521.2	0.18	688.0	0.19
	45		500.5	0.18	660.7	0.18
